# Eukaryotic elongation factor 2 kinase regulates the synthesis of microtubule‐related proteins in neurons

**DOI:** 10.1111/jnc.13407

**Published:** 2015-11-17

**Authors:** Justin W. Kenney, Maja Genheden, Kyung‐Mee Moon, Xuemin Wang, Leonard J. Foster, Christopher G. Proud

**Affiliations:** ^1^ Centre for Biological SciencesUniversity of SouthamptonSouthamptonUK; ^2^ Centre for High‐throughput Biology and Department of Biochemistry & Molecular BiologyUniversity of British ColumbiaVancouverBCCanada; ^3^ Present address: The Hospital for Sick ChildrenProgram in Neuroscience and Mental Health686 Bay St.TorontoON M5G 0A4Canada; ^4^ Present address: South Australian Health & Medical Research Institute and Department of Biological SciencesUniversity of AdelaideAdelaideAustralia

**Keywords:** elongation, mass spectrometry, microtubules, primary neurons, SILAC, translational control

## Abstract

Modulation of the elongation phase of protein synthesis is important for numerous physiological processes in both neurons and other cell types. Elongation is primarily regulated via eukaryotic elongation factor 2 kinase (eEF2K). However, the consequence of altering eEF2K activity on the synthesis of specific proteins is largely unknown. Using both pharmacological and genetic manipulations of eEF2K combined with two protein‐labeling techniques, stable isotope labeling of amino acids in cell culture and bio‐orthogonal non‐canonical amino acid tagging, we identified a subset of proteins whose synthesis is sensitive to inhibition of eEF2K in murine primary cortical neurons. Gene ontology (GO) analyses indicated that processes related to microtubules are particularly sensitive to eEF2K inhibition. Our findings suggest that eEF2K likely contributes to neuronal function by regulating the synthesis of microtubule‐related proteins.

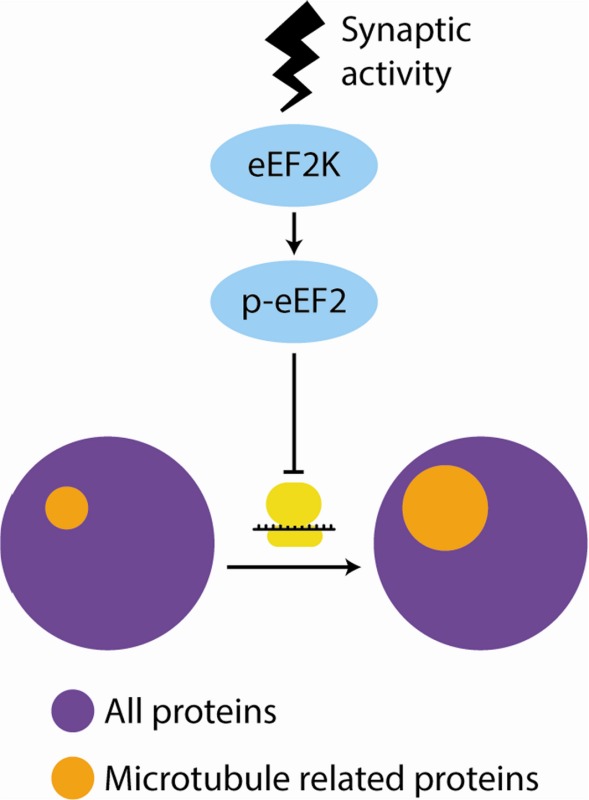

Modulation of the elongation phase of protein synthesis is important for numerous physiological processes in neurons. Here, using labeling of new proteins coupled with proteomic techniques in primary cortical neurons, we find that the synthesis of microtubule‐related proteins is up‐regulated by inhibition of elongation. This suggests that translation elongation is a key regulator of cytoskeletal dynamics in neurons.

**Abbreviations used**
AHA: azidohomoalanine
bic: bicuculline
BONCAT: bio‐orthogonal non‐canonical amino acid tagging
DAVID: database for annotation, visualization, and integrated discovery
eEF2K: elongation factor 2 kinase
GO: gene ontology
NSF: *N*‐ethylmaleimide sensitive fusion protein
SILAC: stable isotope labeling of amino acids in cell culture

The regulation of gene expression at the level of protein synthesis is involved in numerous physiological processes in neurons (Smith *et al*. 2014). Protein synthesis involves three phases, all of which are subject to regulation: initiation, elongation, and termination. Although initiation has generally been considered the most highly regulated step, modulation of translation elongation is increasingly recognized as integral to a variety of functions in both neurons and other cell types (Kenney *et al*. 2014). However, the direct consequences of the modulation of translation elongation on the synthesis of specific proteins remain largely unknown.

The movement of the ribosome along an mRNA during translation elongation is mediated by eukaryotic elongation factor 2 (eEF2). eEF2 is subject to phosphorylation by eEF2 kinase (eEF2K) at Thr56, an event which results in a slowing of elongation (Ryazanov *et al*. 1988; Redpath *et al*. 1993). However, the slowing of elongation has been proposed to result in the increased synthesis of specific proteins. Walden and Thach first reported that low doses of the translation elongation inhibitor, cycloheximide, resulted in an increase in the synthesis of a subset of viral proteins in infected cells, as well as of endogenous proteins in fibroblasts (Walden *et al*. 1981; Walden and Thach 1986). More recently, slowing translation elongation in various neuronal preparations has been reported to increase the synthesis of a small number of proteins related to synaptic plasticity (Scheetz *et al*. 2000; Park *et al*. 2008; Verpelli *et al*. 2010). Furthermore, altering translation elongation *in vivo* affects learning, memory, and plasticity (Park *et al*. 2008; Im *et al*. 2009; Gildish *et al*. 2012; Gold and Wrenn 2012).

To further understand how regulation of translation elongation contributes to neuronal function and physiology, we sought an unbiased method to identify proteins whose synthesis are sensitive to inhibition of eEF2K. To achieve this we combined pharmacological and genetic manipulations of eEF2K with two metabolic protein‐labeling techniques: stable isotope labeling of amino acids in cell culture (SILAC) and bio‐orthogonal non‐canonical amino acid tagging (BONCAT). SILAC allows for both the identification and quantification of changes in the synthesis of specific proteins when used in tandem with mass spectrometry (Schwanhausser *et al*. 2009; Huo *et al*. 2012) and BONCAT allows for the selective isolation of newly synthesized proteins (Dieterich *et al*. 2006; Howden *et al*. 2013; Genheden *et al*. 2015). Through this combination of techniques, we found that levels of microtubule‐related proteins are particularly sensitive to the inhibition of eEF2K.

## Methods

### Materials

All chemicals were from Sigma‐Aldrich (St Louis, MO, USA) unless otherwise noted. The compounds JAN‐452 and JAN–384 were kindly provided by Janssen Pharmaceutica NV (Beerse, Belgium). Primary antibodies were as follows: *N*‐ethylmaleimide sensitive fusion protein (NSF) (2145; Cell Signaling Technology, Danvers, MA, USA), Ppp1 cc (GTX105618; GeneTex, San Francisco, CA, USA), p‐GSK3α/β (9331; Cell Signaling), actin (A2228; Sigma‐Aldrich), eEF2 (Cell Signaling; 2332), and p‐eEF2 (custom‐made by Eurogentec, Liège, Belgium).

### Primary neuronal cell culture

Primary neuronal cortical cultures were isolated from P0 or P1 C57BL/6J mice, or eEF2K‐WT or KO mice of either sex as previously described (Wong *et al*. 2005; Kenney *et al*. 2015). eEF2K mice were generated by Taconic Biosciences, Cranbury, NJ, USA; all mice were bred from colonies maintained at the Southampton General Hospital. All procedures involving mice were done in accordance with United Kingdom Animals (Scientific Procedures) Act 1986 and approved by the University of Southampton.

### SILAC and BONCAT labeling

**Figure 1:**
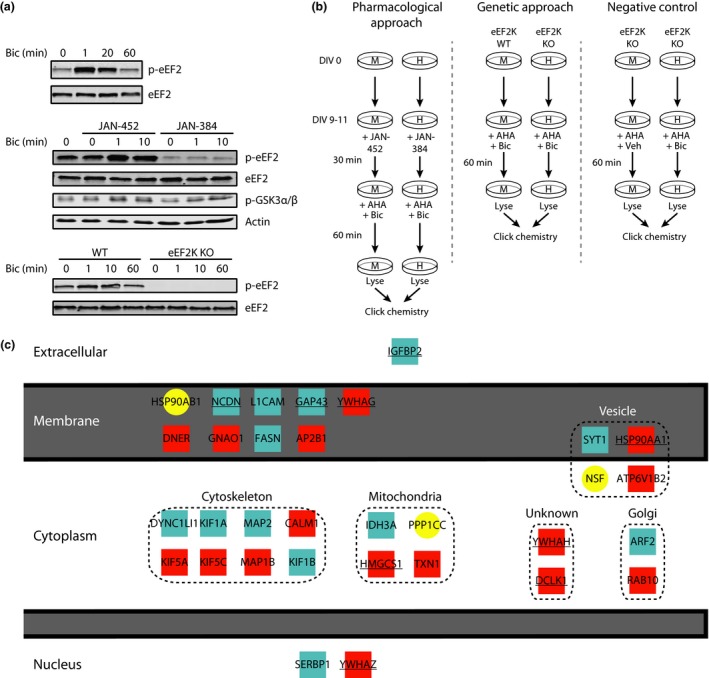
Combining pharmacological and genetic strategies with stable isotope labeling of amino acids in cell culture (SILAC)/bio‐orthogonal non‐canonical amino acid tagging (BONCAT) labeling to identify proteins whose synthesis is regulated by eEF2K. (a) Top, the effect of bicuculline administration on p‐eEF2 levels. Middle, the effect of using an eEF2K inhibitor (JAN‐384) or a less active analog (JAN‐452) on basal and bicuculline‐induced p‐eEF2 and on p‐GSK3α/β. Bottom, examination of the effect of bicuculline on p‐eEF2 levels in neurons from eEF2K‐WT or KO mice. Blots are representative of three to four independent experimental replicates. (b) Experimental designs for combined SILAC/BONCAT labeling to identify proteins whose synthesis are affected by eEF2K inhibition. M and H refer to medium and heavy isotopologs of Arg/Lys, respectively. (c) Summary of proteins whose synthesis was identified as being most sensitive to regulation by eEF2K using a pharmacological approach (blue squares), or a genetic approach (red squares) or both (yellow circles). Underlined proteins were increased in one experiment but not the other. Protein localization was determined using DAVID (Huang *et al*. 2009), or where DAVID lacked annotation, NCBI.

Neurons were plated in customized neurobasal medium (Dundee Cell Products, Dundee, UK) lacking methionine, lysine, and arginine. At plating, methionine was added to the same concentration as in neurobasal and lysine and arginine were substituted with either ‘medium’ Arg (87 mg/mL; ^13^C_6_) and Lys (150 mg/mL; ^2^H_4_) or ‘heavy’ Arg (89 mg/mL; ^13^C_6_+^15^N_4_) and Lys (154 mg/mL; ^13^C_6_+^15^N_2_) all from Silantes. Neurons were plated in 10 cm dishes at high density (~ 1500–2000 cells/mm^2^); four separate independent replicates were used for each set of experiments (Fig. 1b). After 9–11 days *in vitro* neurons were starved of methionine for 30 min and co‐treated with vehicle [dimethylsulfoxide; with non‐isotopic Arg/Lys] or bicuculline (50 μM) and azidohomoalanine (AHA) (2 mM; Bachem, Bubendorf, Switzerland; F‐4265). Compounds JAN‐452 and JAN‐384 (5 μM) were added 30 min prior to application of bicuculline and AHA. Isolation of newly synthesized proteins and preparation for mass spectrometry were performed as described (Genheden *et al*. 2015) except that at the final step peptides were stabilized with 1.0% trichloroacetic acid prior to processing for mass spectrometry.

### Mass spectrometry

Mass spectrometry was performed essentially as described (Huo *et al*. 2012) except that the triazole derivative of Met was considered as a variable modification as in (Somasekharan *et al*. 2012). Median M/H values from experimental replicates were used for analysis. Light Arg/Lys containing peptides were excluded from analysis because of an overabundance of these peptides.

### qRT‐PCR

Neurons were treated with bicuculline (50 μM) or vehicle dimethylsulfoxide for 60 min prior to lysis. JAN‐452 or JAN‐384 was administered 30 min prior to bicuculline/vehicle. RNA was purified from cultures using the GeneJET RNA purification kit (K0731; Thermo Fisher, Waltham, MA, USA) and QIAshredder spin columns (79656; Qiagen, Valencia, CA, USA). One μg of RNA was reverse transcribed using the ImProm‐II reverse transcription system (Promega, Madison, WI, USA). qPCR was performed with 1 μL of cDNA using the GoTaq qPCR master mix (A6001; Promega) on a StepOne real‐time PCR system (Life Technologies, Grand Island, NY, USA). PCR conditions were as follows: 95°C for 2 min followed by 40 cycles of 95°C for 15 s and 60°C for 45 s. Median Ct values from samples run in triplicate were used for analysis using the ΔΔCt method with *Gapdh* as the control. Prior to use in experiments, all primer pairs were determined to be efficient (100 ± 10%) and yield only one appropriately sized product based on melt‐curve analysis and agarose gel electrophoresis. Primers were as follows: *Calm*: 5′‐CTCCGTTCTTCCTTCCTTCG‐3′ and 5′‐TTCCTTGGTTGTGATGGTGC‐3′; *Nsf*: 5′‐ CGCCAGTCCATCATCAATCCCGA‐3′ and 5′‐ACCACAACCAGGGGGTCCGT‐3′; *Ppp1 cc*: 5′‐ GATGTCTTAGGCTGGGGTGA‐3′ and 5′‐TGGCGTTGGGCTTCTTTTTC‐3′; *Kif5a*: 5′‐ CCTCTCCACCAAGAGACAGC‐3′ and 5′‐CAAACAGTCCAACATCACGGG‐3′; *Map2*: 5′‐ AACATTCTGCTGGGGGCGGAAA‐3′ and 5′‐CACCACCTGGCCTGTGACGGA‐3′; *Map1b*: 5′‐ CGGACAGTGCTTTGAGAAC‐3′ and 5‐GTGAGGCTGGCTTTGTTG‐3′; *Gapdh*: 5′‐TGCACCACCAACTGCTTAGC‐3′ and 5′‐GGCATGGACTGTGGTCATGA‐3′; *Arc*: 5′‐TGAGACCAGTTCCACTGATG‐3′ and 5′‐CTCCAGGGTCTCCCTAGTCC‐3′.

### Isolation of newly synthesized proteins and SDS‐PAGE

Neurons were starved of Met for 30 min in Met‐free neurobasal medium and then labeled for 1 h in the presence of 2 mM AHA. After labeling, neurons were washed three times in ice cold phosphate‐buffered saline (PBS) and then lysed [50 mM Tris‐HCl, 50 mM β‐glycerophosphate, 1 mM EGTA, 1 mM EDTA, 1% Triton‐X, 2 ×  protease inhibitors (Roche Molecular Biochemicals, Indianapolis, IN, USA)] on ice. The lysate was centrifuged at 14 000 *g* for 10 min at 4°C and the supernatant was used for click‐chemistry using a biotin‐alkyne (B10185; Invitrogen, Carlsbad, CA, USA) and the protein reaction kit (C10276; Invitrogen) so that only newly synthesized proteins are tagged with biotin. Un‐reacted biotin was removed via gel‐filtration (GE; PD MiniTrap G‐25) using PBS‐T (0.01% Tween) as flow‐through. After washing in PBS‐T, 50 μL of streptavidin magnetic beads (88817; Thermo Fisher, Waltham, MA, USA) were added and incubated overnight at 4°C with rotation to isolate only newly synthesized proteins on the beads. Beads were washed five times in cold PBS‐T and bound proteins were eluted into 20 μL of SDS sample buffer at 95°C for 5 min. Samples (i.e., proteins bound to, and subsequently eluted from, the streptavidin beads) were run on sodium dodecyl sulfate–polyacrylamide gel electrophoresis as previously described (Kenney *et al*. 2015). Nitrocellulose membranes were blocked in Tris‐buffered saline with 0.1% Tween and 2% bovine serum albumin and probed with appropriate antibodies as indicated.

### Statistical analyses

Immunoblot data were quantified using LICOR software (version 3.0, Lincoln, NE, USA). Data were analyzed using two‐way anovas. SPSS version 20.0.0 (IBM, Armonk, NY, USA) was used for analyses.

## Results

### Identification of proteins whose synthesis depends on eEF2K activity

To maximize our ability to detect proteins whose synthesis are sensitive to eEF2K activity, we sought manipulations that would result in large differences in p‐eEF2 levels. The experimental design also took into account the complexity and expense of performing mass spectrometric analyses. To elevate eEF2K activity we used bicuculline, a GABA receptor antagonist (Kenney *et al*. 2015). Bicuculline administration results in a rapid increase in p‐eEF2 that is back to baseline levels after 60 min (Fig. 1a). To reduce eEF2K activity, we employed two complementary strategies: (i) use of a novel and specific eEF2K inhibitor, JAN‐384 (IC_50_ = 5 nM), and comparison of its effects with a less active structural analog, JAN‐452 (IC_50_ > 30 μM) (Versele *et al*. 2014). JAN‐384 is highly selective, having at least an 80–200 fold greater potency toward eEF2K than any others in a screen against 243 kinases (Table 1; Versele *et al*. 2014). JAN‐384 inhibited both the basal and bicuculline‐induced levels of eEF2 phosphorylation, even at early times after bicuculline treatment where eEF2 phosphorylation is maximal and had no effect on the phosphorylation of GSK3α/β (Fig. 1a). (ii) Primary neurons derived from either eEF2K‐WT or KO mice: as expected, neurons derived from eEF2K‐KO mice were completely devoid of eEF2 phosphorylation, and stimulation with bicuculline did not affect this (Fig. 1a).

**Table 1:** Specificity of JAN‐384. The specificity of JAN‐384 was assessed against a panel of 243 protein kinases (Millipore Corporation, Billerica, MA, USA). Those which were affected by JAN‐384 with IC_50_ values within 200‐fold of eEF2K are listed; all others were even less affected by this compound

Kinase	IC_50_ (nM)
eEF2K	5
GSK3a	400
CK2a2	631
GSK3b	631
CDK9cyclinT1	794
238 other kinases	> 1000

To identify proteins whose synthesis is regulated by eEF2K within a short time frame, we combined two metabolic labeling techniques: SILAC and BONCAT (Howden *et al*. 2013; Genheden *et al*. 2015). SILAC allows for the identification and quantification of differences in the synthesis of specific proteins, whereas BONCAT allows for the selective isolation of newly synthesized proteins on a short time scale. To balance the transience of the bicuculline‐induced increase in p‐eEF2 and providing enough material for mass spectrometry, we labeled neurons with AHA for 60 min. We employed three mutually complementary approaches using the combined SILAC/BONCAT technique (Fig. 1b): (i) neurons derived from C57Bl6/J mice were treated with bicuculline and either with JAN‐452 or JAN‐384 (Fig. 1b; left), (ii) neurons derived from eEF2K‐WT or KO mice were both treated with bicuculline (Fig. 1b; middle) and, (iii) as a negative control, neurons derived from eEF2K‐KO mice were treated with either bicuculline or vehicle (Fig. 1b; right). By comparing results from both pharmacological and genetic manipulations we aimed to minimize the limitations inherent in each approach: while pharmacological manipulations allow for more precise temporal manipulations, they may suffer from off‐target effects; whereas genetic manipulations are more targeted, the complete lack of a protein might alter the function of other proteins for which it may be a binding partner.

From both the pharmacological (blue) and genetic (red) approaches, we identified 32 proteins whose synthesis was up‐regulated in an eEF2K‐dependent manner (Fig. 1c; Table 2). A cut‐off of 1 standard deviation above the median M/H ratio was used to define proteins whose synthesis is most sensitive to eEF2K inhibition as this corresponded to a minimum M/H ratio of approximately 2. Three proteins overlapped in the pharmacological and genetic experiments (Fig. 1c; circles). Of the 32 proteins, nine were identified as eEF2K‐sensitive in one type of experiment but not the other (Fig. 1c; underlined). Importantly, there was no overlap between proteins sensitive to eEF2K inhibition and those that were sensitive to bicuculline administration in the absence of eEF2K (i.e., Fig. 1b, right).

**Table 2:** Summary of proteins increased by at least 1 SD above median M/H ratio in experiments outlined in Fig. 1b

Pharmacological approach		Genetic approach	
Protein	M/H ratio	Protein	M/H ratio
SytI	6.89	Calm1	2.93
Ywhag	4.22	Map1b	2.85
Map2	3.51	Dner	2.63
Gap43	3.41	Hmgcs1	2.63
Ppp1 cc	3.12	Gnao1	2.62
L1cam	3.03	Kif5c	2.52
Nsf	2.90	Kif5a	2.52
Ncdn	2.72	Rab10	2.46
Dync1li1	2.70	Ywhah	2.42
Kif1a	2.70	Ppp1 cc	2.32
Hsp90ab1	2.62	Txn1	2.25
Fasn	2.60	Ap2b1	2.21
Kif1b	2.59	Nsf	2.18
Idh3a	2.56	Hsp90aa1	2.13
Igfbp2	2.43	Ywhaz	2.06
Arf2	2.40	Dclk1	2.02
Serbp1	2.40	Atp6v1b2	2.01
		Hsp90ab1	1.99

We performed gene ontology (GO) analysis using the database for annotation, visualization, and integrated discovery bioinformatics tool (ver. 6.7) (Huang *et al*. 2009) using all identified proteins from individual approaches (Fig. 1b) as the background for each set of GO analyses. For both the experiments using the eEF2K inhibitor and those involving the eEF2K WT/KO mice, the most highly enriched GO term among eEF2K‐sensitive proteins was ‘microtubule‐associated complex’ (Table 3). Although not all GO terms reached the generally accepted threshold (*p* < 0.05) for statistical significance, in both experiments, GO terms related to protein localization and movement were consistently amongst the most enriched. Furthermore, there was no overlap of GO terms with those identified in the negative control experiment (i.e., eEF2K‐KO vs. KO; Fig. 1b, right). Taken as a whole, these findings suggest that the translation of mRNAs encoding microtubule‐related proteins is particularly sensitive to eEF2K inhibition.

**Table 3:** Top 7 enriched gene ontology (GO) terms from different experimental approaches (Fig. 1b) identified using DAVID (Huang *et al*. 2009). CC, cellular component; MF, molecular function; BP, biological process. Reported *p*‐values are EASE/modified Fisher's exact *p*‐values calculated by DAVID

Pharmacological approach			Genetic approach			Negative control		
GO description	Fold enrichment	*p*	GO description	Fold enrichment	*p*	GO description	Fold enrichment	*p*
Microtubule‐associated complex (CC)	7.87	0.043	Microtubule‐associated complex (CC)	6.67	0.058	Antioxidant activity (MF)	12.61	0.016
Microtubule motor activity (MF)	7.69	0.045	Protein domain specific binding (MF)	5.09	0.030	Response to inorganic substance (BP)	12.59	0.001
Calmodulin binding (MF)	6.59	0.061	Melanosome (CC)	3.66	0.069	Response to reactive oxygen species (BP)	12.59	0.016
Identical protein binding (MF)	6.59	0.061	Pigment granule (CC)	3.66	0.069	Response to oxidative stress (BP)	8.39	0.007
Synapse (CC)	5.24	0.027	Intracellular protein transport (BP)	3.49	0.082	Cellular ion homeostasis (BP)	6.30	0.017
Microtubule (CC)	3.31	0.091	Cellular protein localization (BP)	3.26	0.097	Chemical homeostasis (BP)	6.30	0.017
Neuron projection (CC)	3.03	0.054	Establishment of protein localization (BP)	2.91	0.064	Cellular chemical homeostasis (BP)	6.30	0.017

### eEF2K‐dependent changes in protein synthesis are unlikely to be due to changes in mRNA expression

**Figure 2:**
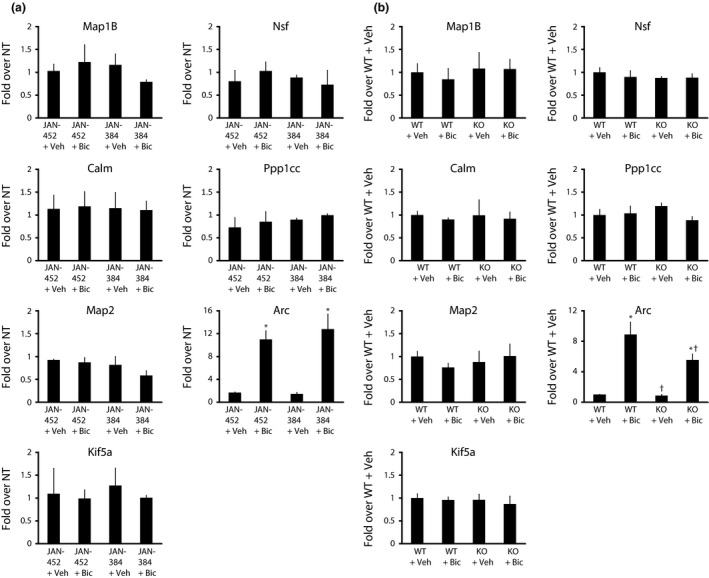
Neither genetic nor pharmacological inhibition of eEF2K affects total levels of mRNAs for most eEF2K targets. (a) Total levels of specific mRNAs in neurons administered JAN‐452 or JAN‐384 in the presence of bicuculline or vehicle dimethylsulfoxide (DMSO) compared to untreated neurons (NT); *n* = 3. (b) Total mRNA levels from neurons derived from eEF2K‐WT or KO mice and treated with bicuculline or vehicle; *n* = 3. *p <* *0.05 (main effect of bicuculline), ^†^ *p *<* *0.05 (main effect of eEF2K KO) via 2 × 2 (bicuculline × eEF2K inhibitor/eEF2K KO) anova.

To determine if some of the particularly interesting targets were regulated at the transcriptional or translational levels, we examined the levels of their mRNAs. As a positive control for the effects of bicuculline, we examined *Arc* transcription as *Arc* translation has previously been found to be up‐regulated by eEF2K in a transcriptionally independent fashion (Park *et al*. 2008). There was no significant effect of inhibiting eEF2K with JAN‐384 on the total levels of mRNA for all genes examined (Fig. 2a; all 2 × 2 (inhibitor × bicuculline) anovas, *p*'s > 0.05). As expected, we found that bicuculline induced a large increase in *Arc* mRNA (*F*(1, 3) = 192.1, *p *<* *0.001) with no significant effect of JAN‐384 on this (Fig. 2a).We examined the same panel of mRNAs in eEF2K‐WT and KO mice and also found little effect of genetic removal of eEF2K on mRNA levels (Fig. 2b; all 2 × 2 anovas, *p*'s> 0.05; except for *Arc*). Interestingly, in addition to identifying a main effect of bicuculline on *Arc* transcription (*F*(1, 3) = 246.1, *p *<* *0.001), we found a main effect of the eEF2K‐KO on the amount of *Arc* (*F*(1, 3) = 5.8, *p* = 0.043). This suggests that the constitutive eEF2K knockout may affect transcription (at least for this gene) differently from the transient inhibition of eEF2K by JAN‐384.

### Confirmation of SILAC/BONCAT targets

**Figure 3:**
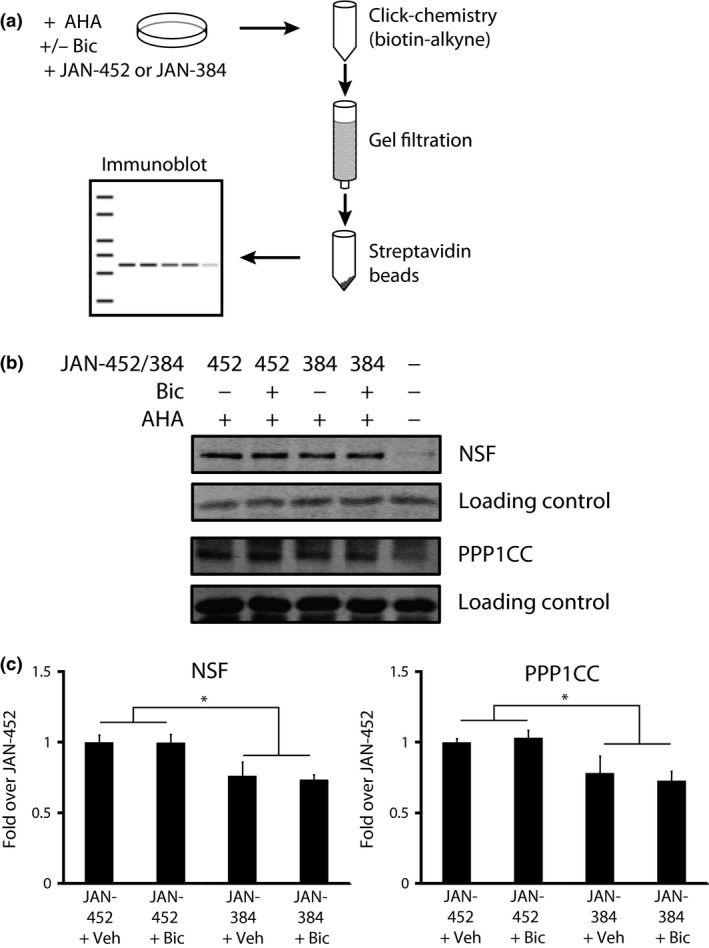
Confirmation of two eEF2K targets identified via stable isotope labeling of amino acids in cell culture (SILAC)/bio‐orthogonal non‐canonical amino acid tagging (BONCAT). (a) Experimental design for labeling, isolating, and identifying newly synthesized proteins. (b) Representative western blots for *N*‐ethylmaleimide sensitive fusion protein (NSF) and PPP1CC. (c) Quantification of western blot data. *p <* *0.01 via 2 × 2 (bicuculline × eEF2K inhibitor) anova;* n* = 8–9.

We used a focused method to confirm our mass spectrometric findings (Fig. 3a). There was very little background from cells that had not been labeled with AHA (Fig. 3b; ‐AHA lane). As a ‘loading control’, we used a non‐specific band at approximately 25 kDa that was apparent on all gels. We found that JAN‐384 decreased the amount of both newly synthesized NSF (*F*(1, 3) = 14.4, *p*  =  0.001) and PPP1CC (*F*(1, 3) = 11.2, *p = *0.002) (Fig. 3c). Surprisingly, there was no effect of bicuculline alone on the synthesis of either NSF or PPP1CC. This suggests that eEF2K activity may be more important for maintaining their basal rates of synthesis rather than regulating their production in response to elevated synaptic activity. Nonetheless, these data do confirm our mass spectrometry findings that inhibiting eEF2K results in a decrease in the amount of newly synthesized NSF and PPP1CCC. The number of proteins we could confirm using this technique was limited by the lack of availability of antibodies that worked in this assay.

## Discussion

Using a novel combination of SILAC and BONCAT, we have conducted the first unbiased large‐scale screen for proteins whose synthesis is regulated by eEF2K activity, allowing us to identify a subset of such proteins. Importantly, we found consistency in the effects of pharmacological and genetic manipulations of eEF2K, with both indicating a role for eEF2K in the regulation of processes related to microtubules and intracellular trafficking. Furthermore, because we examined changes over a short time period (60 min) and there are no alterations in the expression of the mRNAs for several candidates, these changes are unlikely to be related to alterations in transcription or mRNA stability. Instead, they likely reflect alterations in the translation of these mRNAs.

Targeted techniques have previously identified a small number of eEF2K‐regulated proteins related to synaptic plasticity such as activity‐regulated cytoskeleton‐associated protein (ARC), MAP1B, CAMKIIα, and brain‐derived neurotrophic factor (BNDF) (Scheetz *et al*. 2000; Davidkova and Carroll 2007; Park *et al*. 2008; Verpelli *et al*. 2010). Using our unbiased mass spectrometry‐based technique, we also found that synthesis of MAP1B was sensitive to eEF2K inhibition [the other proteins were not detected in any of the three experiments presented (Fig. 1b); however, ARC and CAMKIIα were detected and found to be sensitive to eEF2K inhibition in a preliminary experiment (unpublished observations)]. This points to one of the fundamental limitations of mass spectrometry versus sequencing‐based techniques, that is, that low abundance proteins may not be detected. However, the strength of mass spectrometry is that we are directly examining newly synthesized proteins instead of inferring the translation of their mRNAs as in techniques such as ribosome profiling (Ingolia *et al*. 2011). Ribosome profiling suffers from a particular drawback when studying mRNAs regulated through the control of elongation, as mRNAs bound to ribosomes which are stalled during elongation will actually be scored as actively translated messages.

In the central nervous system, modulation of eEF2K function has been consistently linked to changes in synaptic plasticity and learning and memory (Belelovsky *et al*. 2005; Park *et al*. 2008; Im *et al*. 2009; Gildish *et al*. 2012; Taha *et al*. 2013). However, the mechanism underlying the effect of altered eEF2K activity in these contexts has remained obscure. One potential mechanism is suggested from the work of Verpelli *et al*. (2010) who found that eEF2K is important for dendritic spine stability. Here, we find that eEF2K regulates the expression of several proteins involved in microtubule function and trafficking. Recent work has found that microtubules undergo rearrangement following learning and glutamate receptor activity (Fanara *et al*. 2010) and that microtubule function is critical for memory formation (Uchida *et al*. 2014). Although microtubules are largely thought to be restricted to mediating intracellular transport in dendrites and axons, there are also complex interactions with the actin cytoskeleton that play a key role in regulating dendritic spine morphology (Gu *et al*. 2008; Hu *et al*. 2008; Jaworski *et al*. 2009). Thus, findings from this study suggest that eEF2K may play an important role in learning and memory by modulating dendritic spines via alterations in the synthesis of microtubule‐related proteins.

The mechanism by which altering protein synthesis at elongation results in mRNA‐specific translational regulation remains unknown. It has been suggested that decreasing elongation rates allows poorly initiating mRNAs to more successfully compete against other mRNAs for a limited pool of initiation factors (Walden and Thach 1986; Belelovsky *et al*. 2005). Another possibility is that the proportion of common and rare codons may confer translational control at the elongation phase since rare codons are translated more slowly than common codons (Gustafsson *et al*. 2004). However, we found no correlation between sensitivity to eEF2K inhibition from our SILAC/BONCAT data and codon frequency (unpublished observations). Our identification of additional proteins whose synthesis is sensitive to eEF2K inhibition should provide important clues for delineating the mechanism that confers mRNA‐specific translational control via modulation of elongation.
